# Survive or swim: different relationships between migration potential and larval size in three sympatric Mediterranean octocorals

**DOI:** 10.1038/s41598-020-75099-1

**Published:** 2020-10-22

**Authors:** Katell Guizien, N. Viladrich, Á. Martínez-Quintana, L. Bramanti

**Affiliations:** 1 CNRS-Sorbonne Université, Laboratoire d’Ecogéochimie des Environnements Benthiques, LECOB, Observatoire Océanologique de Banyuls Sur Mer, 1 avenue Pierre Fabre, 66650 Banyuls sur Mer, France; 2 Institute of Environmental Science and Technology (ICTA), Universitat Autònoma de Barcelona (UAB), Building C Campus UAB, 08193 Bellaterra (Cerdanyola del Vallès), Barcelona Spain; 3 Institut de Ciències del Mar, ICM-CSIC, Passeig Marítim de la Barceloneta, 37-49, 08003 Barcelona, Spain; 4 Present Address: Department of Environment and Sustainability, University at Buffalo, Buffalo, NY 14260 USA; 5 Present Address: Department of Geology, University at Buffalo, Buffalo, NY 14260 USA

**Keywords:** Ecology, Animal migration, Behavioural ecology

## Abstract

Knowledge about migration potential is key to forecasting species distributions in changing environments. For many marine benthic invertebrates, migration happens during reproduction because of larval dispersal. The present study aims to test whether larval size can be used as a surrogate for migration potential arising from larval longevity, competence, sinking, or swimming behavior. The hypothesis was tested using larvae of three sympatric gorgonian species that release brooded lecithotrophic larvae in the same season: *Paramuricea clavata*, *Corallium rubrum* and *Eunicella singularis*. Despite different fecundities and larval sizes, the median larval longevity was similar among the three species. Free-fall speed increased with larval size. Nevertheless, the only net sinkers were the *P. clavata* larvae, as swimming was more common than free fall in the other two species with larger larvae. For the other two species, swimming activity frequency decreased as larval size increased. Interestingly, maximum larval longevity was lowest for the most active but intermediately sized larvae. Larval size did not covary consistently with any larval traits of the three species when considered individually. We thus advise not using larval size as a surrogate for migration potential in distribution models. The three species exemplified that different mechanisms, i.e., swimming activity or larval longevity, resulting from a trade-off in the use of energy reserves can facilitate migration, regardless of life history strategy.

## Introduction

Forecasting the impact of climate change on biodiversity requires mechanistic models similar to those used for climate change predictions (IPBES 2030 work program, https://ipbes.net/o4-supporting-policy). Species, habitat or niche models should incorporate the mechanisms regulating species spatial distributions to project future changes in biodiversity and species richness. Since Darwin’s early observations, the ecological niche concept has been key to explaining the spatial distribution of species under selection pressures that arise from the environment and interactions between species^1,2^. The concept postulates that each species should have a unique set of conditions defining its persistence. Correlative approaches linking species occurrence data with environmental descriptors have been extensively applied to infer species niches and to project species spatial distributions (reviewed in ^3^). However, correlative approaches might fail to predict changes in species spatial distributions when environmental conditions evolve more rapidly than the species can cope with^4^. Indeed, when local environmental conditions change, a species’ persistence will depend on its ability to track suitable conditions for existence through migration and/or to adapt locally^5^.

Trait-based mechanistic models explicitly include migration processes through a migration parameter, i.e., expansion distance per unit time (meta-population^6^; meta-community^7^). However, spatiotemporal mechanistic models are still limited in their application due to knowledge gaps in migration parameters^8^. For sessile species such as plants in terrestrial ecosystems or several benthic invertebrates in marine ecosystems, migration happens during reproduction because of offspring dispersal. Estimating offspring dispersal distance in such species is challenging because reduced larval size limits their tracking. Propagule size has been used as a proxy of dispersal distance, such as smaller seeds dispersing farther^9^. In the ocean, other proxies have been used to infer larval dispersal distance. For example, it is common to assume that lecithotrophic (nonfeeding) larvae disperse less than planktotrophic (feeding on plankton) larvae^10,11^ or that different reproductive strategies impact dispersal potential, such as less fecund internal brooders dispersing less than more fecund broadcast spawners^12,13^. However, in contrast to the passive dispersal of seeds, larvae can display motility behavior and regulate their buoyancy during dispersal^14^. Larval motility behavior adds a degree of freedom in the hyperspace of larval traits defining the larval dispersal potential of a species. Hence, larval behavior may obscure the aforementioned putative proxy relationships between dispersal potential and reproductive strategy and/or larval size.

At sea, offspring migration parameters, i.e., dispersal distance per generation, have been inferred indirectly from population genetic^15^, otolith and shell microchemistry^16^, or/and biophysical modeling^17^ data. In addition to intrinsic methodological uncertainties^18^, population genetics and otolithometry estimates suffer the same forecasting limitation as correlative species distribution models, as they result from the past history of species-environment interactions^19^. Biophysical modeling is the only tool that enables the prediction of offspring dispersal distances in future environmental scenarios, but it requires incorporating larval traits that regulate dispersal. These larval traits are release timing and location, pelagic larval duration (PLD, the period of time during which larvae of a benthic species can disperse in the flow until settlement) and motility behavior that results from the balance between free fall and swimming activity^20^. Minimum PLD corresponds to larval competence onset, which is defined as the moment in which pelagic larvae can metamorphosize in their benthic form to settle. However, PLD can extend as long as some larvae survive and are still able to metamorphosize. Certainly, flow dispersal can delay settlement opportunities in the benthic habitat, and because dispersal distance often increases with PLD^21^, migration potential should instead be assessed using the upper limit of the PLD range.

In the present study, we selected three sympatric sessile species differing slightly in reproductive strategies, which depend on lecithotrophic larvae of different sizes to migrate. Larval traits regulating their dispersal and hence offspring migration parameters were quantified. The white gorgonian *Eunicella singularis* (Esper, 1794) is a species with an approximately 25-year life expectancy^22^. It is distributed throughout the western Mediterranean and Adriatic Sea and occasionally present in the eastern Mediterranean, dwelling on rocky bottoms in shallow waters, as well as on coralligenous formations in deeper sublittoral waters^23,24^. Each year, *E. singularis* releases 1–5 lecithotrophic ciliated larvae (planulae) per polyp between the end of June and early July^25,26^. *E. singularis* planulae are internally brooded, symbiotic, and bright pink, with a major axis of ~ 2.5 mm and a minor axis of 0.5 mm^22^. The Mediterranean red coral *Corallium rubrum* (Linnaeus, 1758) is a slow-growing species with presumably more than a 100-year life expectancy^27^. It is distributed throughout the Mediterranean Sea and adjacent Atlantic coasts between 20 and at least 600 m depth^28^. Each year, *C. rubrum* releases less than 2 lecithotrophic ciliated larvae (planulae) per polyp between the end of July and early August^29^. *C. rubrum* planulae are internally brooded and do not host symbiotic algae. They are white and club-shaped, with a major axis of ~ 1 mm and a minor axis of 0.3 mm^30^. *C. rubrum* planulae can survive up to 42 days in the water column (median larval longevity of 32 ± 11 days), showing negative buoyancy with free-fall speeds between 0.03 and 0.09 cm s^−1^ and a high swimming activity frequency (82 ± 14.5%^31^). Finally, the red gorgonian *Paramuricea clavata* (Risso, 1826) is a species with a more than 30-year life expectancy and is widely distributed along the western basin of the Mediterranean Sea and in the Adriatic Sea^32^. *P. clavata* spawns 8 to 20 oocytes per polyp every year in two short release events at 15-day intervals in June^33^. Fertilization is external, and embryogenesis takes place on the surface of the colony between 48 and 72 h upon oocyte release (surface brooder). Fertilized oocytes become lecithotrophic ciliated planulae within 48 h after fertilization. *P. clavata* planulae do not host symbiotic algae, are purple and can reach 1 mm in length when completely developed^34^. Earlier studies based on qualitative observations suggested that *P. clavata* larvae have low dispersal potential and should settle near parental individuals^33^.

The present study aims to test whether larval size can be used as a surrogate for the migration potential arising from larval dispersal. Larval traits required to forecast larval dispersal in biophysical modeling were quantified for *P. clavata* and *E. singularis,* as similarly done for *C. rubrum*^31^. In particular, we assessed release timing, PLD (derived from larval longevity and metamorphosis rate), buoyancy, and larval vertical motility behavior. Differences in these larval traits were tested among the three species. The observed relationships between larval traits and larval size are compared to those expected from either a life history strategy or an energetic perspective.

## Material and methods

### Larval collection

Larval release of *P. clavata* and *E. singularis* was recorded between 2011 and 2016 off the Catalan coast. *P. clavata* (surface brooder) released oocytes between June 20th and July 14th, while *E. singularis (*internal brooder) released larvae between June 15th and July 26th (Table 1, Supplementary Material 1). Offspring of the two species were collected in 2011, 2012, 2014 and 2016 at two sites along the Catalan coast (Cap de Creus, Spain and Banyuls-sur-Mer, France). *P. clavata* oocytes were collected with 50 ml syringes from the surface of at least 10 different females in each site and within 2 days upon release. Two days after collection, 36% ± 5% (mean ± standard deviation, SD) of collected oocytes had transformed into planula larvae. Larvae were kept in 2 L containers filled with filtered seawater (< 5 µm) at 18–20 °C (average temperature in June-July at a 27 m water depth in Banyuls Bay—Service d'Observation du Laboratoire Arago). *P. clavata* larvae were reared in dark conditions, as they do not host symbiotic algae. Water was renewed every other day. To obtain *E. singularis* planulae*,* 10 female colonies were collected at each site and transferred to closed-circuit aquaria at 18–20 °C with oxygenation until planula release (end of June^26^). Released planulae were collected daily for 20 days. Larvae released within two consecutive days were pooled to form age cohorts. *E. singularis* larvae contain Symbiodiniaceae dinoflagellates, whose metabolism is activated by light^35^. To test the possible benefit of autotropic input for larval longevity and/or metamorphosis, each cohort was separated into two groups: one group kept in dark conditions and one group exposed to a 12 h photoperiod (12 h light/12 h dark cycle). All larvae were maintained at 18–20 °C in 2 L containers with filtered seawater (< 5 µm), which was renewed every other day.

The following traits were quantified in the laboratory: (1) larval survival rate (proportion of initial larval number that survived in the absence of predation), (2) metamorphosis rate (proportion of the initial larvae that metamorphosed into polyps), (3) surface area of larvae (maximum projected planar surface area of larvae in photographs), (4) larval body density (larval mass per unit volume), (5) free-fall speed (fall speed of nonswimming larvae in still seawater at 20 °C), and (6) swimming activity frequency (proportion of time during which larvae are actively swimming). Different traits were measured between 2011 and 2016 (Table 1, Supplementary Material 1).

### PLD: larval survival and metamorphosis rates in the absence of predation

PLD is defined by the overlap of two periods of time: the period during which metamorphosis is possible (competence window) and the period during which the larvae survive (larval longevity). Thus, PLD was defined after assessing larval survival and metamorphosis rates. For larval survival and metamorphosis rate assessment, ~ 400 larvae were randomly chosen from each release event and subdivided into three 0.4 L glass containers (replicates, n = 100, 130 or 150) with filtered seawater (< 5 µm), which was renewed every other day to simulate the conditions of pelagic dispersal. Metamorphosis was defined as the moment at which larvae underwent morphological differentiation into a tiny eight-tentacled polyp^22,34^. For *P. clavata*, larval survival and metamorphosis rates were quantified on larvae from one release event in 2014 and three release events in 2016. Such a procedure was adopted to test the effects of both inter- and intra-annual variability. For *E. singularis*, larval survival and metamorphosis rates were quantified on larvae from three release events in 2016. Larvae from one release event were maintained in dark conditions, and larvae from the other two release events were maintained in a 12 h photoperiod. Healthy larvae and polyps from both studied species were counted every other day (precision of 3%, estimated from repeated counts) while pipetting them into a different container for water renewal. Damaged larvae were also transferred but not counted. Sometimes, *P. clavata* polyps were detected a few days after their metamorphosis due to their small size and potential confusion with damaged larvae. The larval survival rate at a given larval age was calculated as the number of healthy larvae of that age divided by the initial number of larvae at age 0. The metamorphosis rate at a given larval age was calculated as the number of polyps at a given larval age divided by the initial number of larvae at age 0. For each release event (n = 3), minimum, half and maximum larval longevity were defined as the time since larval release at which survival rates were 95%, 50% and 5%, respectively. The effect of the release event on the minimum, half, and maximum larval longevity and metamorphosis rate was tested by one-way ANOVA (MATLAB command “anova1”, MATLAB 2012) with “Release event” as the factor (4 levels and 3 replicates for *P. clavata*, 2 levels and 3 replicates for *E. singularis* in dark/light conditions). When a significant difference was found, a post hoc test was performed (MATLAB command “multcompare” with a 5% significance level for Tukey’s honestly significant difference (HSD) criterion, MATLAB 2012).

### Surface area, body density and free-fall speed of inactive larvae

The projected planar surface of 160 < 12-day-old larvae of *P. clavata*, *E. singularis* and *Corallium rubrum* was measured using scaled pictures. Scaled larval pictures were taken in top-view Petri dishes with a SONY DFW-X700 camera mounted on a binocular, using VISILOG software for image scaling (for *C. rubrum*, pictures were taken during the previous study^31^). Pictures were processed with a routine developed by the authors with the MATLAB Image Processing toolbox that semiautomatically detects larvae after optimizing image contrast and binarizing it (MATLAB 2012, SurfaceObjetFinal.m routine available at https://github.com/guizien/Larval-actography; the main steps are detailed in Supplementary Material 2). Body density and free-fall speed of planulae of *P. clavata* and *E. singularis* were quantified for different ages (2-day age cohorts). Measurements for the same age with planulae from different 2016 release events were considered replicates. The buoyancy of planulae was defined as the difference between planula body density and seawater density. It could be positive, negative or neutral, leading to larvae that float, sink or stay in position while inactive, respectively. In this study, the modified dual-density method described in Ref.^36^ was used to estimate the specific body density of planulae, which consisted of observing whether they floated or sank through a series of 7 density gradients produced by layering seawater (density of 1.0265 g ml^−1^ at 20 °C) either over a sucrose solution (distilled water plus sucrose) with densities ranging from 1.027 to 1.039 g ml^−1^ or below a sucrose solution of 1.024 g ml^−1^ density. An additional test tube containing only seawater was used to confirm the buoyancy under nearly natural conditions with a seawater temperature of 20 °C for *P. clavata* (maximum seawater temperature below a 20 m depth) and 22 °C for *E. singularis* (maximum seawater temperature at an upper 20 m depth).

To immobilize the larvae without killing them while maintaining their shape and their free-fall properties, the larvae were anesthetized by immersing them for less than 15 min in menthol-saturated seawater. Anesthetized larvae were checked under a stereomicroscope, and those with visible damage on their external surface were rejected. Twenty anesthetized larvae were injected sequentially into each test tube and were allowed to settle for 10 min into the density gradient produced by the two fluids. Only the larvae that sank to the bottom were considered denser than the corresponding sucrose solution. The experiment duration was calibrated by checking larval segregation stability in tubes with different sucrose concentrations after different times from 5 min to 1 h. We did not observe larval damage by the sucrose solution with up to 15 min of experiment duration. This procedure was carried out every day until the larvae were approximately 30 days old. A frequency distribution of body density was built for each day, reporting the proportion of larvae denser than each sucrose solution (n = 20 larvae). The 20% and 80% quantiles of these frequency distributions were used to define the range of larval body density values.

For both species, free-fall experiments were carried out at different larval ages until they reached 30 days old. *P. clavata* larvae were not anesthetized, as they displayed almost negligible swimming activity (see “Larval motility behavior: swimming activity frequency”). In contrast, *E. singularis* larvae were anesthetized to measure free-fall speed in the absence of swimming activity. For each species and larval age, a minimum of 30 larvae were gently injected into a settling device filled with seawater at room temperature. The settling device was a square bottle (9 cm width, 20 cm height) topped with a wide funnel (15 cm diameter) to minimize the movement inside the bottle caused by the injection itself. Individual larval free-fall speed was calculated from video recordings after detecting larvae and reconstructing their individual tracks with two semiautomated routines developed by the authors with the MATLAB Image Processing toolbox (MATLAB 2012, Extract_larvae_position_Final.m and Build_track_Final.m routines available at https://github.com/guizien/Larval-actography; the main steps are detailed in Supplementary Material 2). Free fall was recorded over a 5 cm height in the middle of the square bottle to avoid wall effects^37^. Seawater temperature varied among the experiments from 19 to 23 °C, which could have altered free-fall speed measurements. Thus, free-fall speed measurements were split into 2 groups according to experimental temperature (19–21 °C and 21–23 °C). The effect of larval age was tested on both the average and the SD of free-fall speed for the 19–21 °C group only. One-way ANOVAs were performed with 3 levels corresponding to three age groups, namely, fewer than 10 days, between 10 and 20 days and more than 20 days, and with more than 30 replicates for each level (MATLAB command “anova1”, MATLAB 2012). When a significant difference was detected, a post hoc test was performed (MATLAB command “multcompare” with a 5% significance level for Tukey’s HSD criterion, MATLAB 2012). The linear correlation between age and average free-fall speed measured at 19–21 °C was also tested (MATLAB command “corrcoef”, MATLAB 2012).

### Larval motility behavior: swimming activity frequency

Swimming activity frequency was used to quantify larval motility behavior. Swimming activity frequency was defined as the proportion of time in which larvae were actively swimming and thus did not settle or free fall. Briefly, it was quantified from larval motion video recordings of fixed duration, dividing the cumulative duration of swimming larval tracks by the maximum potential tracking duration if all larvae had been swimming during the recording (number of larvae multiplied by recording duration). For each larval motility behavior assay, 20 larvae of the same age were randomly chosen and divided into 2 groups (n = 10 each) and injected into 2 different 50 ml flat-faced plastic containers (38 × 60 × 20 mm) to limit interference by individual larval movement^37^. Larval motility behavior was recorded for 90 min at 25 frames per second with a digital camera (SONY DCR-SR78). A small distance between the front and back walls (20 mm) was chosen (1) to constrain larvae to vertical motion and (2) to maintain them in the focal plane of the camera so that each individual could be tracked. Assays were performed simultaneously in the two containers and considered replicates. The 90 min recording was divided into six successive sequences of 15 min during which larvae were exposed alternately to cold white light (CWL, 6500 K, PAR = 480 µmol photons m^–2^ s^–1^) and infrared (IR) light (PAR < 1 µmol photons m^–2^ s^–1^). This procedure was designed to study the sensitivity of swimming activity to light exposure as experienced at sea with depth variations during larval dispersal^20^. Recording started 2 min after the last larva was injected, ensuring that at the beginning of each recording, flow motion due to injection had disappeared. Larvae were kinesthetically stimulated using a pipette to agitate water in the container where larvae were stored just before transferring them to the small experimental container. To obtain approximately the same level of kinesthetic stimulation for all the replicates, a similar number of pipette injections were applied each time. The effect of such stimulation is expected to decay with time. For both species, swimming activity frequency was quantified at least every other day (more often when possible) until the larvae were 20 days old. Larvae assayed at different ages did not necessarily come from the same release event. Swimming activity frequency was calculated for each of the two groups of 10 larvae (replicates, n = 2), in each 15 min sequence and at each larval age in both species. Given that larvae assayed in the different 15-min slots at the same age and in the same larval maintenance photoperiod condition were the same, the effects on swimming activity frequency of (1) kinesthetic stimulation, (2) light conditions and (3) age and photoperiod conditions during larval maintenance in the absence of kinesthetic stimulation and for the same light conditions were evaluated separately^20^. First, the effect of kinesthetic stimulation was tested by comparing the swimming activity frequency at different times after kinesthetic stimulation was stopped. To do so, a paired t-test was performed between the mean swimming activity frequency measured at different larval ages (n = 25) during the three 15-min slots of CWL (immediately, 30 min after and 1 h after kinesthetic stimulation was stopped). Second, the effect of light condition on swimming activity frequency was tested considering only the last four 15-min slots, more than 30 min after kinesthetic stimulation stopped. To do so, a paired t-test was performed between the mean swimming activity frequency measured at different larval ages (n = 25) during the last two 15-min slots of CWL and the last two 15-min slots of IR. Third, the effects of larval age and photoperiod conditions during larval maintenance on swimming activity frequency were tested together considering only the last four 15-min slots, more than 30 min after kinesthetic stimulation stopped. Two-way ANOVAs were performed for each light condition separately, with the 2 replicates per level being the two vials in the 30-min slots with the same light condition, ensuring replicate independence (Factor 1: 6 levels (7/9/11/13/15/17-day age) x Factor 2: 2 levels (with/without photoperiod during larval maintenance); MATLAB command “anovan”, MATLAB 2012).

## Results

### PLD: larval survival and metamorphosis rates in the absence of predation

**Figure 1:**
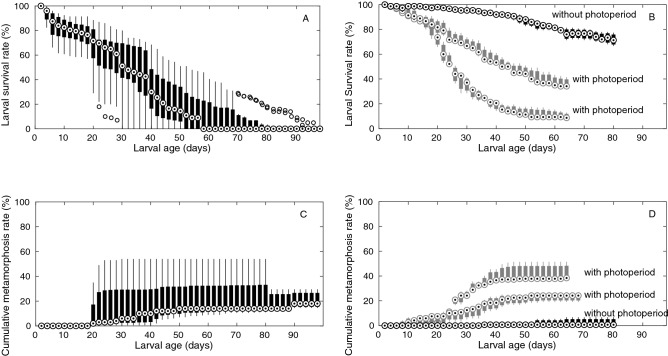
Larval survival rate of *P. clavata* for 90 days (**A**) and *E. singularis* for 65 days (**B**) and polyp metamorphosis rate for *P. clavata* for 90 days (**C**) and *E. singularis* for 65 days (**D**). Each boxplot depicts the median value (circled dot), the 25th and 75th percentiles (bar), the minimum and maximum outlier values (solid lines) and outliers (open circles) of the rates measured every 2 days. For *P. clavata*, boxplots display statistics after grouping four assays of three replicates carried out with larvae reared in dark conditions. For *E. singularis*, boxplots display statistics for each assay of three replicates taken separately, two of them carried out on larvae reared with photoperiod and one on larvae reared in dark conditions.

The survival of *P. clavata* larvae was highly variable at all ages (large boxplot heights, Fig. 1A). Pooling all release events and locations, the minimum larval longevity was 3.5 ± 1.5 days (mean ± standard deviation, SD), the median larval longevity was 32 ± 11 days, and the maximum larval longevity was 64 ± 22 days. Neither minimum nor maximum larval longevity was significantly different between release events (ANOVA: F_3,8_ < 2.3, p > 0.15) due to the high variability among replicates. Only the median larval longevity was significantly different between release events. In Banyuls-sur-Mer, the median larval longevity was higher than that in Cap de Creus for the 2016 release events; however, it was similar to that in Cap de Creus for the 2014 release event (ANOVA: F_3,8_ = 8.4, p < 0.05, Tukey's HSD: T2 < T4 and T3 < T4). Metamorphosis rates were not significantly different among release events or locations at any age (ANOVA: F_1,2_ < 4, p > 0.07). Thus, metamorphosis rates were grouped for all release events and locations. Extensive metamorphosis was detected after 20 days and continued until the larvae were 70 days old. The cumulative metamorphosis rate ranged from 10.5 to 54% of the initial larval pool after 70 days, with a median of 14% (Fig. 1C).

For *E. singularis*, larval survival was strongly affected by light treatment: larval survival was significantly higher in the dark than in the light treatment after larvae were 4 days old (ANOVA: F_1,4_ > 10.4; p < 0.05). Minimum larval longevity was 30 ± 5.3 days in the dark treatment and 7.7 ± 2.3 days in the light treatment. Median larval longevity was 35 ± 11.6 days in the light treatment, while it could not be determined in the dark treatment (> 78 days, experiment duration). Moreover, larval survival varied significantly for larvae older than 20 days between release events within the same brood (ANOVA: F_1,4_ > 10.4; p < 0.05 comparing the two release events in the light treatment, Fig. 1B). Finally, the maximum larval longevity was > 78 days (experiment duration) in both treatments. In the light treatment, metamorphosis started after 6 days, but the daily rates remained low (< 4%) until 20 days and were similar between the two release events (ANOVA: F_1,4_ < 7.7; p > 0.05). The cumulative metamorphosis rate increased abruptly from 10 to 40% between 20 and 40 days for larvae from one of the two release events in the light treatment, resulting in a significant difference between the two release events after 26 days (Fig. 1D, ANOVA: F_1,4_ > 13.5; p < 0.021). Metamorphosis yielded a median of 37% of the initial larval pool in one release event and 24% in the other after 40 days. Metamorphosis was observed until all larvae had died. In the dark treatment, metamorphosis was delayed until larvae were 25 days old, and it accumulated to less than 7% of the initial larval pool at 78 days.

### Surface area, body density and free-fall speed of inactive larvae

**Figure 2:**
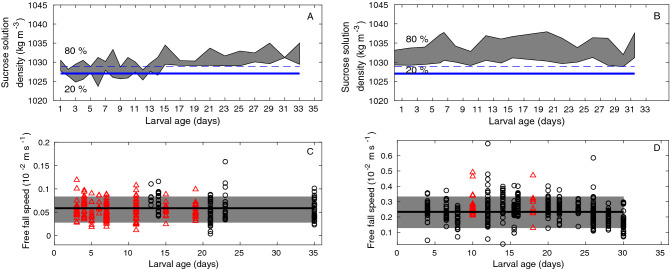
The 80% and 20% quantiles of larval body density of *P. clavata* (**A**) and *E. singularis* (**B**) are displayed by the gray areas during the first 30 days of larval survival. The thick (dashed) line indicates seawater density at 38 psu and 20 °C (12 °C). Individual free-fall velocities of *P. clavata* (**C**) and *E. singularis* (**D**) larvae measured in seawater at 19–21 °C (black circle) and 21–23 °C (red triangle) in different reproductive events. Mean sinking velocity values (solid line) and 80% confidence intervals around the mean (gray area) estimated from linear regression on the 19–21 °C data.

Larval surface areas were 0.16 ± 0.03 mm^2^ (mean ± SD) for *P. clavata,* 0.29 ± 0.07 mm^2^ for *C. rubrum* and 0.61 ± 0.21 mm^2^ for *E. singularis.* The majority (70%) of the anesthetized larvae of *P. clavata* and all *E. singularis* larvae were denser than seawater at 20 °C until they reached 30 days old (Fig. 2A,B). *P. clavata* larval body density ranged from 1025 to 1033 kg m^−3^ for young larvae (< 15 days old) and 1029–1034 kg m^−3^ for larvae up to 30 days old, while for *E. singularis* larvae, it ranged from 1030 to 1035 kg m^−3^ at all ages (i.e., denser than seawater even at 12 °C). Notably, up to 80% of the young *P. clavata* larvae (< 15 days old) were less dense than seawater at 12 °C. The larval body density of both species was variable (5 kg.m^-3^ difference among 12 different larvae), which correlated with large interindividual variability in free-fall speeds (SD of approximately 35% of the mean, Fig. 2C,D). Free-fall speed measured at 20 °C was not linearly correlated with age (R^2^ = 0.73 and p = 0.16 for *P. clavata*; R^2^ = 0.22 and p = 0.41 for *E. singularis*), and when larval ages were grouped into classes of 10-day intervals, the free-fall speeds among classes were not significantly different (ANOVA: F_1,3_ = 7.8 and p = 0.07 for *P. clavata*; F_2,13_ = 3.2 and p = 0.07 for *E. singularis*) in either of the two species. Moreover, the free-fall speed at 20 °C was not significantly different for larvae less than 10 days old, between 10 and 20 days old and more than 20 days old (ANOVA: F_1,3_ = 2.2 and p = 0.23 for *P. clavata*; F_2,13_ = 1.4 and p = 0.28 for *E. singularis*). Thus, free-fall speed can be summarized by its mean ± SD for each species, being 0.056 ± 0.021 cm s^-1^ for *P. clavata* and 0.23 ± 0.08 cm s^-1^ for *E. singularis* (Fig. 2C,D).

### Larval motility behavior: swimming activity frequency

**Figure 3:**
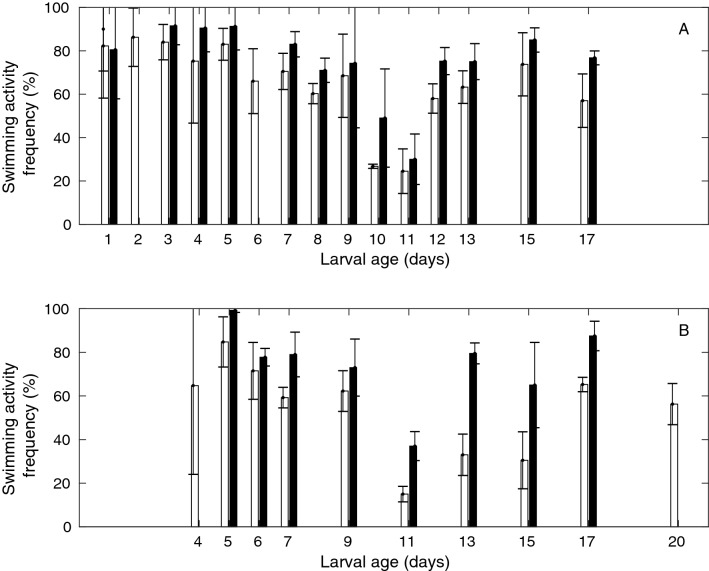
Mean swimming activity frequency of 2 groups of 10 larvae (black—in dark conditions—and white—in light conditions—bars) of *E. singularis* during the first 17 days of larval survival. (**A**) Larvae maintained with a 12 h photoperiod; (**B**) Larvae maintained in the dark for 17 days.

*P. clavata* larvae spent 95% of the time on the bottom of the containers at 20 °C and at all larval ages tested in the motility experiment (up to 20 days old). During the 5% of time remaining, larvae exhibited crawling behavior or free fall. Additionally, *P. clavata* larvae did not exhibit any swimming behavior throughout the study (up to 100 days old). In contrast, *E. singularis* larvae were active swimmers. Indeed, despite having a negative buoyancy at 20 °C, most larvae were found in the center of the containers during larval survival experiments (up to 80 days old). Swimming activity frequency was highly variable, ranging from 0 to 100%, depending on the 15 min sequence analyzed. A consistent increase of 10% in swimming activity after the first 15-min sequence of the motility experiment was detected, indicating that kinesthetic stimulation tended to inhibit larval swimming activity in *E. singularis*. Under cold white light conditions, swimming activity frequency was significantly lower during the first 15-min sequence (CWL1) of each experiment than during the second (CWL2) and third sequences (CWL3; paired t-test, CWL1 vs CWL2: t_25_ = − 4.7, p < 10^–4^; CWL1 vs CWL3: t_25_ = − 4.8, p < 10^–4^). However, 30 min after the kinesthetic stimulation stopped, swimming activities among CWL sequences were no longer different. Thus, only the 15-min sequences after 30 min from the beginning of the experiment (two in CWL and two in IR) were used and pooled together to address the effect of light exposure, larval age, and photoperiod conditioning during larval maintenance. Swimming activity frequency varied between 20 and 90% (Fig. 3A,B). A significant effect of light exposure was found with a higher swimming activity frequency in dark conditions (75%) than under light exposure (62%; paired t-test, CWL vs IR: t_20_ = − 6.6, p < 10^–4^).

A significant effect of larval age and photoperiod conditioning during larval maintenance was found on the swimming activity frequency under light exposure (2-factor ANOVA: F_1,17_ = 8.2 and p = 10^–2^ for photoperiod conditioning during larval maintenance; F_5,17_ = 6.9 and p = 10^–3^ for larval age). Under dark conditions, however, only age had a significant effect on swimming activity frequency (2-factor ANOVA: F_1,17_ = 0.01 and p = 0.92 for photoperiod conditioning during larval maintenance; F_5,17_ = 9.5 and p = 2 10^–4^ for larval age). Post hoc tests indicated that swimming activity frequency was significantly lower for larvae maintained without a photoperiod than for those maintained with a photoperiod in the light exposure sequences and for 11-day-old larvae than for larvae of all other ages in both light and dark exposure sequences. This latter effect surpassed the effects of all other factors, as the average swimming activity frequency was 68 ± 23%, except when larvae were 11 days old, when it dropped to 26 ± 11.3%.

### Comparison of larval traits among *P. clavata*, *C. rubrum* and *E. singularis*

**Figure 4:**
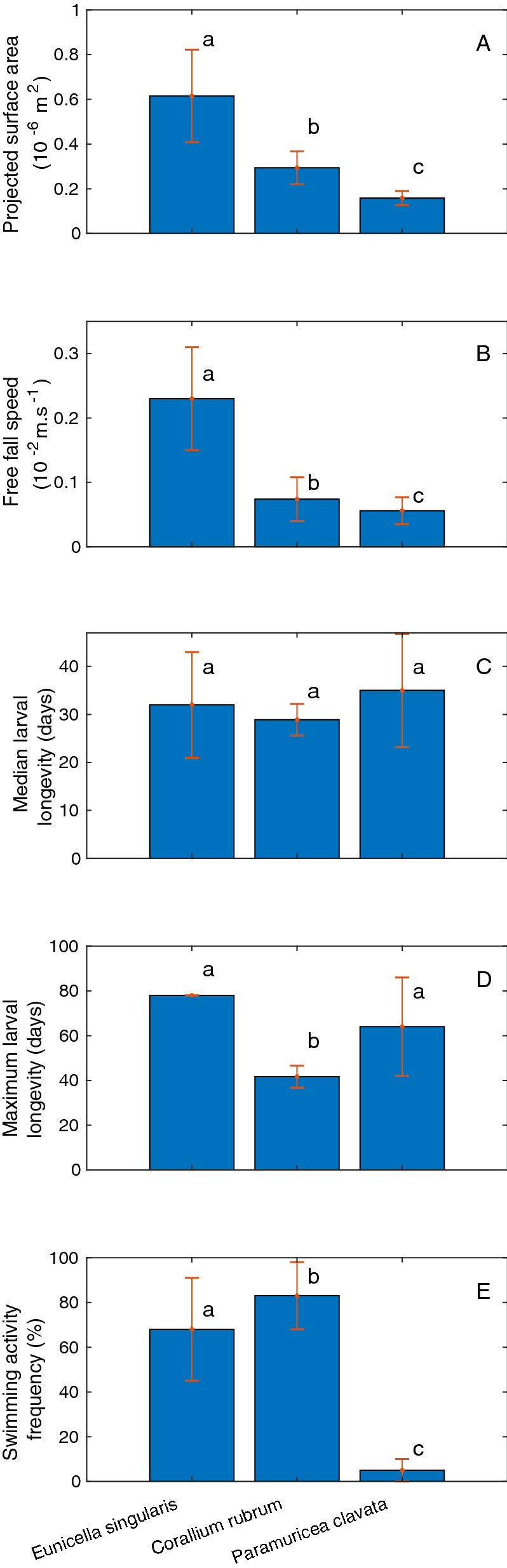
Larval projected surface area (**A**), free-fall speed (**B**), median larval longevity (**C**), maximum larval longevity (**D**) and swimming activity frequency (**E**) for three sympatric species: *C. rubrum* (Cr, internal brooder), *E. singularis* (Es, internal brooder with symbionts), and *P. clavata* (Pc, external brooder). Different letters (a, b, c) display significant differences. The same letter for different species indicates that the difference was not significant.

Larval surface area was significantly different among the three species, with *E. singularis* larvae being twice as large as *C. rubrum* larvae and four times larger than *P. clavata* larvae (*E. singularis* > *C. rubrum* > *P. clavata*; ANOVA: F_2,536_ = 589; p < 0.001, Fig. 4A). The mean free-fall speed at 20 °C was also significantly different, with the same trend (*E. singularis* > *C. rubrum* > *P. clavata*; ANOVA: F_2,1058_ = 1031; p <  < 0.01, Fig. 4B), despite large interindividual variation in free-fall speed and larval body density. Median larval longevity (larval age at 50% survival) was similar among the three species (*E. singularis* = *C. rubrum* = *P. clavata*; ANOVA: F_2,24_ = 0.8; p = 0.45, Fig. 4C), whereas maximum larval longevity was significantly greater for *E. singularis*, followed by *P. clavata* and *C. rubrum* (ANOVA: F_2,24_ = 10.9; p < 0.01, Fig. 4D). Finally, swimming activity frequency was also significantly different among the three species, but in this case, *C. rubrum* larvae were the most active, followed by *E. singularis* and *P. clavata* (ANOVA: F_2,426_ = 658; p <  < 0.01, Fig. 4E).

## Discussion

The three octocoral species (*E*. *singularis*, *P. clavata* and *C. rubrum)* produce lecithotrophic planula larvae and share the same median larval longevity of 30 days (in temperature and salinity conditions close to those under natural conditions at sea). A similar larval longevity has been reported for other octocoral species in the Red Sea, regardless of being brooders or broadcast spawners^38^. Such long larval longevity contrasts with the short duration or even absence of precompetency, which is assumed to characterize the brooding strategy. Brooder invertebrates have been reported to display short minimum PLDs, generally of a few days (Mediterranean Sea: Porifera^39^; tropical corals^40^; Pacific Ocean: bryozoans^41^), while the minimum PLD commonly extends up to 30 days for broadcast spawners belonging to different taxa due to late competency (echinoid larvae^36^; other taxa^42^). In the present study, metamorphosis was observed before median larval longevity was reached for *E. singularis* and *P. clavata* (after approximately 20 days). A recent study indicated that metamorphosis happened even earlier in the larval stage for *E. singularis* (8 days) and *P. clavata* (11 days)^43^. However, in the same study^43^, metamorphosis of *C. rubrum* larvae was only observed after 27 days, a duration similar to their median longevity, suggesting that competence, and hence minimum PLD, might not be related to a brooding or spawning reproductive strategy.

Nevertheless, in biophysical modeling, the minimum PLD defined by competency is related to the lower range of dispersal distances^17,21^. The upper range of dispersal distances, defining the maximum migration potential in one generation when flow conditions do not allow for settlement, should instead be related to median or maximum PLD. In the present study, larvae of the three species remained competent until they died, meaning that the factor limiting PLD was larval longevity. Among brooding species releasing lecithotrophic larvae, a trade-off between larval size and larval longevity can be expected. On the one hand, from an ecophysiological perspective, smaller larvae with smaller energy reserves should survive less and not delay settlement with respect to larger larvae. As a consequence, smaller larvae are expected to disperse shorter distances (desperate larva hypothesis^44,45^). We thus first question whether smaller larvae disperse for shorter durations than larger larvae. On the other hand, from a life history strategy perspective, species with larger larvae, which are expected to be less fecund than species with smaller larvae, should display a higher reproductive success to persist^11^. Thus, because offspring losses increase with dispersal duration due to longer exposure to predation^46^, the inverse hypothesis also deserves to be considered: do larger larvae produced by less fecund species disperse for shorter durations than smaller larvae produced by more fecund species (to ensure higher survival during the larval phase)?

Among the three species studied, the premise of the life-history argument (the lower the fecundity is, the larger the larvae^47^) was only partly valid, meaning that maternal investment was not the same among the three species^48,49^. While the species with the smallest larvae was the one with the highest fecundity (*P. clavata*, major axis < 1 mm, 8–13 oocytes polyp^−1^,^33^)*,* the species with the lowest fecundity (*C. rubrum,* major axis ~ 1 mm, less than 2 larvae polyp^−1^,^50^) was not the one with the largest larvae (*E. singularis,* major axis ~ 2.5 mm, 1–5 larvae polyp^-1^,^25,51^). The only larval trait that covaried with species fecundity was swimming activity frequency, but the relationship was the opposite of that expected from the life-history strategy perspective. The larvae of the species with the highest fecundity (*P. clavata)* hardly swam and thus settled, which is expected to limit dispersal duration by rapid settlement, notwithstanding specific flow conditions. In contrast, the larvae of the species with the lowest fecundity (*C. rubrum)* swam most of the time, and much longer than they free fell, which is expected to facilitate their dispersal. In fact, the only factor that could constrain the migration potential of *C. rubrum* was maximum larval longevity. However, maximum larval longevity did not limit the dispersal potential of the other low fecundity species, *E. singularis*. Finally, none of the traits that are expected to regulate larval dispersal (PLD, swimming activity frequency) was consistent with the postulate “the lower the fecundity is, the lower the larval dispersal potential” when examined individually. Therefore, larval dispersal distances cannot be inferred from life-history strategy alone, meaning that the latter should not be used as a proxy of dispersal capability.

Therefore, could ecophysiological traits be more appropriate for inferring dispersal? Do smaller larvae have lower dispersal capabilities than larger ones?

The only larval trait that decreased with larval size was free-fall speed. When larvae are passive, they behave as sedimentary particles^52^, and larger larvae with larger free-fall speeds settle faster and disperse shorter distances than smaller larvae. However, in the case of active larvae, swimming activity could compensate for free fall. In the present study, the species with larger larvae (*E. singularis* and *C. rubrum*) avoided sinking by active swimming. In contrast, the species with the smallest larvae (*P. clavata*) did not swim and settled quickly in the absence of flow motion, a mechanism expected to limit its dispersal. Smaller larvae, despite their slower free-fall speeds, would disperse less than larger larvae due to the active swimming behavior of the latter, probably supported by larger energy reserves^53^. Nevertheless, lecithotrophic larvae have limited energy reserves upon release, and energy consumption might alter larval traits, such as buoyancy, as reported in *C. rubrum* (decrease in free-fall speed with age^31^).

Ultimately, energy reserve consumption could alter survival as well, which likely explained the reduced maximum larval longevity of *C. rubrum* compared to *P. clavata.* In such a case, anticipating actual larval dispersal potential in ocean flow between the two species is not straightforward and requires larval dispersal simulations^21^. While free fall leads to rapid settlement in horizontal flows and de facto limits dispersal duration, settlement due to free fall would be delayed under coastal upwelling^54^. In such flow conditions (frequently observed in the western Mediterranean Sea, where the species dwells^55^), a longer biological pelagic duration would enable farther dispersal. The combination of nonswimming behavior and a long pelagic duration in *P. clavata* can lead to very contrasting effective dispersal in environments differing in flow conditions. This is consistent with the various spatial scales at which genetic differences were reported in *P. clavata* population genetic studies^56,57^. In fact, depending on coastal flow structure, *P. clavata* larval traits allowing larval transport for 10 days are likely to result in a wide range of dispersal distances^21^. For this species with reduced free fall, avoiding swimming minimized energy consumption, and extended larval longevity. Free-fall with long larval longevity might have been an efficient trade-off that enabled *P. clavata* population recovery after mass mortality events and inbreeding avoidance^58,59^ despite high self-recruitment in low-flow areas^56,57^*.* In contrast, the migration potential of *C. rubrum* is less consistent with the genetic differentiation observed at a half-meter-square spatial scale^60^. A median larval longevity of 30 days, a precompetence period of 27 days^43^ and the high swimming activity of *C. rubrum* conflict with such genetic population structure, as the transport of neutrally buoyant larvae after 15 days can reach 100 km^61^. Factors other than limited dispersal capabilities can reduce gene flow and could be invoked to explain such population genetic structuring of *C. rubrum*^62^. For instance, demographic factors such as a low fecundity^50^ combined with effective population size reduction due to harvesting of larger colonies^27^ should be considered.

In the case of *E*. *singularis,* the relationships between larval traits, energy consumption and dispersal potential are unclear. Neither free-fall speed nor larval body density changed with age while the larvae sustained active swimming throughout the study. This suggests that the symbiotic relationship of *E*. *singularis* with *Symbiodinium* spp*.* may sustain larval energy needs. However, the longevity of *E. singularis* larvae maintained with a 12 h photoperiod was lower than that of larvae maintained in dark conditions, suggesting that light may not always increase larval dispersal potential in symbiotic species^63^. Light oxidative damage may threaten larval longevity^64^. A more precise assessment of metabolism and energy content throughout the larval stage would test the role of symbionts throughout this stage. In any case, the photoperiod conditions in which we maintained *E. singularis* larvae were designed to mimic natural conditions. *E. singularis* larvae can be expected to maintain themselves in the upper part of the water column because of their swimming behavior and thus to be exposed to light irradiance.

The similar longevities between large *E. singularis* larvae reared with a photoperiod and small *P. clavata* larvae reared in dark conditions suggests a trade-off between the cost of active swimming behavior combined with light oxidative stress during dispersal and the amount of energy reserves given upon release. However, large larvae, being generally less numerous than small ones upon release^47^ (*E. singularis* has a lower fecundity than *P. clavata*), should better cope with pressures acting between release and recruitment than small ones to compensate for their lower initial number. Quantifying predation rates in plankton remains challenging^65^. Prey selection by zooplanktivores is often positively correlated with prey size^66^. However, other traits regulating prey/predator encounters, such as their respective densities^67^ and motilities^68^, are also key to determining predation rates. The only documented process that supports the trade-off between larval size and larval number is postsettlement success: higher postsettlement survival of larger larvae compared to small ones could compensate for their lower initial number^69^.

In summary, the three brooder species displayed different life-history strategies, with both fecundity and larval size varying by a factor of 5. All larval traits (maximum larval longevity, free-fall speed and swimming activity frequency) except median larval longevity also differed among the three species, but no proxy relationship was found between any larval traits (except free-fall speed) and either fecundity or larval size. In addition, none of the larval traits assessed could explain species migration potential when considered alone. Nevertheless, trade-offs between the larval traits regulating a species’ dispersal were consistent with the consumption of energy reserves upon release.

## Supplementary information

Supplementary Information
