# Supplementary material for: Survive or swim: different relationships between migration potential and larval size in three sympatric Mediterranean octocorals
Source: Sci Rep. 2020 Oct 22;10:18096. doi: 10.1038/s41598-020-75099-1 (PMC7581755; doi:10.1038/s41598-020-75099-1)
Supplement: Supplementary file 1 — Supplementary Information [file 41598_2020_75099_MOESM1_ESM.docx]

**Survive or swim : different relationships between migration potential and larval size in three sympatric Mediterranean octocorals**

K. Guizien^1^, N. Viladrich^1,2^, Á. Martínez-Quintana^3, 4^, L. Bramanti^1^

^1^ CNRS-Sorbonne Université, Laboratoire d'Ecogéochimie des Environnements Benthiques, LECOB, Observatoire Océanologique de Banyuls sur Mer, 1 avenue Pierre Fabre - 66650 Banyuls sur Mer (FRANCE).

^2^ Institute of Environmental Science and Technology (ICTA), Universitat Autònoma de Barcelona (UAB), Building C Campus UAB, 08193 Bellaterra (Cerdanyola del Vallès), Barcelona (SPAIN).

^3^ Institut de Ciències del Mar, ICM-CSIC, Passeig Marítim de la Barceloneta, 37-49, 08003 Barcelona (SPAIN).

^4^ currently, Department of Environment and Sustainability and Department of Geology, University at Buffalo, Buffalo, NY 14260, USA

Corresponding Author: Katell Guizien

Current address: CNRS-Sorbonne Université, Laboratoire d'Ecogéochimie des Environnements Benthiques, LECOB, Observatoire Océanologique de Banyuls sur Mer, 1 avenue Pierre Fabre - 66650 Banyuls sur Mer (FRANCE).

Email: guizien@obs-banyuls.fr

**Supplementary material 1 Release timing and Sampling dates**

|  |  |  | LARVAL TRAITS ASSESSED | | | | | |
| --- | --- | --- | --- | --- | --- | --- | --- | --- |
| Species | Release date | Site | Larval survival rate | Metamorphosis rate | Larval body density | Free fall speed | Swimming activity frequency | Swimming speeds |
| ***Paramuricea clavata*** | 21/06/2011 | CC | **\|** | **\|** | **\|** | **\|** | **X** | **X** |
|  | 22/06/2012 | CC | **\|** | **\|** | **\|** | **\|** | **X** | **X** |
|  | 05/07/2013 | CC | **\|** | **\|** | **\|** | **\|** | **\|** | **\|** |
|  | 7 to 10/07/2013 | CC | **\|** | **\|** | **\|** | **\|** | **\|** | **\|** |
|  | 14/07/2013 | CC | **\|** | **\|** | **\|** | **\|** | **\|** | **\|** |
|  | 22/06/2014 | CC | **X** | **\|** | **\|** | **\|** | **\|** | **\|** |
|  | 20 to 22/06/2016 | CC | **X** | **X** | **X** | **X** | **\|** | **\|** |
|  | 23/06/2016 | B | **X** | **X** | **X** | **X** | **\|** | **\|** |
|  | 13/07/2016 | B | **\|** | **\|** | **\|** | **\|** | **\|** | **\|** |
| ***Eunicella singularis*** | 15 to 16/06/2012 | CC | **\|** | **\|** | **\|** | **\|** | **X** | **X** |
|  | 19 to 21/07/2012 | CC | **\|** | **\|** | **\|** | **\|** | **X** | **X** |
|  | 22 to 24/06/2016 | B | **X** | **X** | **X** | **X** | **\|** | **\|** |
|  | 05 to 11/07/2016 | B | **X** | **X** | **X** | **X** | **\|** | **\|** |
|  | 15 to 20/07/2016 | B | **\|** | **\|** | **X** | **X** | **\|** | **\|** |
|  | 22 to 26/07/2016 | B | **\|** | **\|** | **\|** | **\|** | **\|** | **\|** |

**Table 1:** Release timing recorded off the Catalan coast (CC= Cap de Creus, B=Banyuls-sur-mer) for the two gorgonian species since 2011 (for *Eunicella singularis,* in 2012 and 2016 only*)* and larval traits assessed when larvae were sampled in the release timing.

**Supplementary material 2 Image processing routines**

**Semi-automated larvae projected planar surface measurements**

A routine was developed with the Matlab Image Processing toolbox to help automate measurements of larvae projected planar surface from larvae images including an embedded scale (Matlab2012b, routine SurfaceObjetFinal.m, available at <https://github.com/guizien/Larval-actography>). The main routine entry is a Red Green Blue graphics file displaying larvae together with any reference distance enabling to scale the image. Other entries are the color plane used to detect the larvae (Red/Green/Blue, in which the larvae best contrast from the background), image format (e.g. 4:3 / 16:9, according to the settings used when taking pictures of the larvae which define pixel distortion), image orientation (rotation angle of 0 or 90 degrees to be applied to the image), grayscale (0 if larvae are lighter than background / 1 if larvae are darker than background), minimum surface of larvae (minimum surface area in mm^2^ used to threshold larval projected surface area), maximum larval size (radius in pixel of the morphing function used to average background, should be bigger than the size of the object the routine should detect), minimum larval size (number of pixels used to threshold any detected object after binarization, only objects larger than this threshold are kept), gray level (upper value of the range in which contrast will be increased), connectivity (connectivity value required in selected object after binarization), scale orientation (horizontal or vertical scale orientation as displayed after image rotation), scale size (value in metric unit of the reference distance). Output of the routine is a variable containing as many surface area values in square metric units given in entry, as larvae detected on the input image.

The 10 steps of the routine are :

1) Uploading graphics file

2) Computing image scaling from reference distance manual selection

3) Cropping image manually to remove unnecessary part of the image

4) Extraction of the selected color plane and inverting the greyscale so that background turns to darkest colors

5) Computing image background applying a low-pass 2D filtering over disks which radius is defined by maximum larval size

6) Substracting image background to original image

7) Increasing image contrast by mapping the values in intensity of image to new values such that 1% of data is saturated at low and high intensities of the input range 0 to gray level.

8) Binarization of the image after using the Otsu's method, which chooses the threshold to minimize the intraclass variance of the black and white pixels.

9) Larvae detection as white objects bigger than the minimum larval size given as an entry and displaying the connectivity given as an entry. In two-dimensional image, connectivity can be either 4 (pixels are connected if their edges touch) or 8 (pixels are connected if their edges or corners touch).

10) Detected larvae are labeled, their area in pixel is computed and only those bigger than the minimum larval surface given as an entry are kept (remove artefactual light reflection or shadows detected as objects in step 9) and scaled in square metric unit.

**Semi-automated larval tracks measurements**

Larval motion was recorded at 25 frames per second with a digital camera (SONY DCR-SR78); larval tracks were reconstructed applying the particle-tracking routine developed by the authors with the Matlab Image Processing toolbox to a sequence of frames sampled at a 0.5 s rate (Matlab2012b, routines Extract_larvae_position_Final.m and Build_track_Final.m available at https://github.com/guizien/Larval-actography).

The main steps in the routines are as follows:

On each frame of a sequence, all larvae were detected applying steps 3 to 9 of the routine above. In step 10, detected white objects were limited to a maximum surface of 90 pixels to be considered as larva instead of applying a minimum surface because artefactual light reflections or shadows were mainly detected as large objects in the video context. Each larva track was then reconstructed from nearest positions of two larvae in two successive frames. When this reconstruction was ambivalent for the presence in the frame of more than one larva near to another in the previous frame, the track was stopped and a new track started for each larva. Larval tracks were scaled in pixels using independent vial width for each film. It yield an average spatial resolution for position tracking of 0.0087 cm pixel^-1^ (0.0126 cm pixel^-1^, respectively) on the horizontal (on the vertical, respectively) for *Eunicella singularis* larvae and of 0.0070 cm pixel^-1^ (0.011 cm pixel^-1^, respectively) for *Paramuricea clavata* larvae. A maximum vertical velocity threshold of 1.5 cm s^-1^ was applied which exceeds the free fall or swimming speeds of the larvae of both species.

**Free fall speed measurements**

In free fall experiments, vertical velocity components were computed along each recorded track and filtered out to detect uniform motion (when weight minus buoyancy equals drag) by removing accelerated or decelerated motion as result of the injection of the larvae or when the larvae were settling in the bottom of the containers. A larva is considered in uniform fall motion when the maximum deviation between the mean fall speeds during two consecutive 2.5 s long periods was lower than the standard deviation of the fall speed within the 2.5 s long periods. The individual free fall speed and its precision was determined for each track, hence for each larva, as the mean and standard deviation of the vertical speed during uniform fall motion.
